# Is CD133 Expression a Prognostic Biomarker of Non-Small-Cell Lung Cancer? A Systematic Review and Meta-Analysis

**DOI:** 10.1371/journal.pone.0100168

**Published:** 2014-06-18

**Authors:** Hong Wu, Xiao-wei Qi, Guang-ning Yan, Qing-bi Zhang, Chuan Xu, Xiu-wu Bian

**Affiliations:** 1 Institute of Pathology and Southwest Cancer Center, Southwest Hospital, Third Military Medical University, and Key Laboratory of Tumor Immunopathology, Ministry of Education of China, Chongqing, China; 2 Department of Oncology, Chengdu Military General Hospital, Chengdu, Sichuan, China; 3 Breast Disease Center, Southwest Hospital, Third Military Medical University, Chongqing, China; 4 Department of Public Health, Luzhou Medical College, Luzhou, Sichuan, China; University of North Carolina School of Medicine, United States of America

## Abstract

**Background:**

The clinical and prognostic significance of CD133 in non-small-cell lung cancer (NSCLC) remains controversial. To clarify a precise determinant of the clinical significance of CD133, we conducted a systematic review and meta-analysis to evaluate the association of CD133 with prognosis and clinicopathological features of NSCLC patients.

**Methods:**

The electronic and manual searches were performed through the database of Pubmed, Medline, Web of Science, Scopus, and Chinese CNKI (from January 1, 1982 to January 1, 2014) for titles and abstracts by using the following keywords: “CD133”, “ac133” or “Prominin-1”, and “lung cancer” to identify the studies eligible for our analysis. Meta-analysis was performed by using Review Manager 5.0 and the outcomes included the overall survival and various clinicopathological features.

**Results:**

A total of 23 studies were finally included, and our results showed that CD133 level was significantly correlated with the overall survival (OR = 2.25, 95% CI: 1.24–4.07, *P* = 0.008) of NSCLC patients but not with the disease free survival (OR = 1.33, 95% CI = 0.77–2.30, *P* = 0.31). With respect to clinicopathological features, CD133 level was positively correlated with lymph node metastasis (OR = 1.99, 95%CI = 1.06–3.74, *P* = 0.03), but not correlated with the histological classification (OR = 1.00, 95%CI = 0.81–1.23, *P* = 0.99(ac), OR = 0.87, 95%CI = 0.61–1.24, *P* = 0.45(sc)), or differentiation (OR = 0.94, 95%CI 0.53–1.68, Z = 0.20, *P* = 0.84 random-effect) of NSCLC patients.

**Conclusion:**

High level of CD133 expression trends to correlate with a worse prognosis and a higher rate of lymph node metastasis in NSCLC patients, revealing CD133 as a potential pathological prognostic marker for NSCLC patients.

## Introduction

Lung cancer is the first leading cause of death worldwide. Because of its high incidence and recurrence rate, the 5-years overall survival has been estimated at nearly 10% [1]. According to the theory of cancer stem cells (CSCs), tumors can be recognized as a result of abnormal organogenesis driven by a small subpopulation of cancer cells that are able to be self-renewal and to produce the heterogeneous lineages of cancer cells [2]–[4]. The cell surface marker CD133, also known as prominin-1, is widely used as a CSCs marker in various tumors, including leukemia, lung cancer, colon cancer and brain cancer [5]–[10]. Furthermore, its prognostic and clinicopathological values in the above-mentioned cancers have been widely studied [11]–[14].

Up to date, controversy exists concerning the correlation between CD133 and prognostic value with respect to Non-Small-Cell Lung Cancer (NSCLC) [1], [15]. Some studies reported that high expression of CD133 was correlated with unfavorable prognosis [1], [11], which were hardly concurred by others [16], [17]. Here, we performed a systematic review and meta-analysis in the published literatures to clarify whether the expression of CD133 was associated with the clinicopathological features and prognosis of NSCLC patients.

## Materials and Methods

### Literature Search Strategy

**Figure 1:**
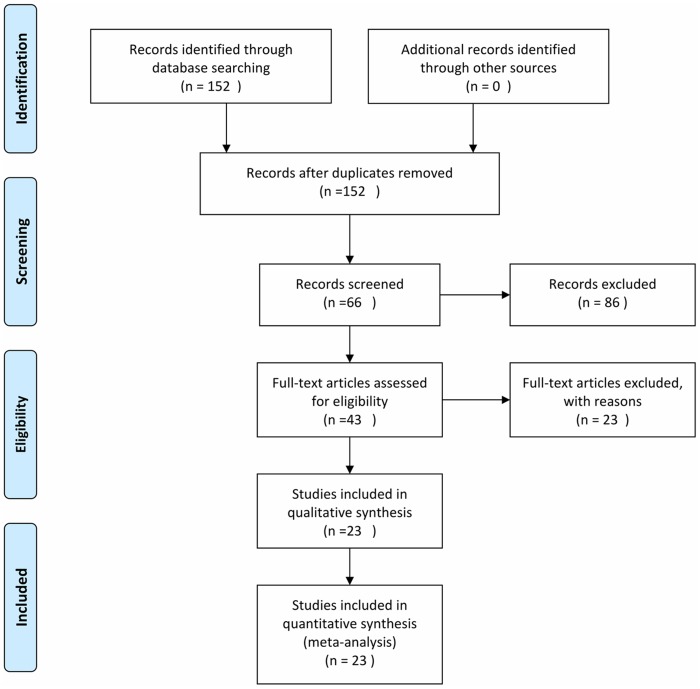
Flow chart for selection of studies.

We adapted the Cochrane Central Register of Controlled Trials, and performed a comprehensive publication search through the Pubmed, Medline, Web of Science, Scopus, and Chinese CNKI database by using the following search strings: “CD133”, “ac133” or “Prominin-1”, and “lung cancer” from January 1, 1982 to January 1, 2014. Titles and abstracts were reviewed to identify reports which examined the association of CD133 level with clinical outcomes, such as overall survival (OS), disease free survival (DFS), and clinicopathological features including age, histology, differentiation and lymph node metastasis in NSCLC patients (Figure 1).

### Selection Criteria

The criteria for inclusion were listed as follows: (1) studies dealing with NSCLC patients (excluding the small cell lung cancer patients); (2) articles concerning about the association of CD133 level with either prognostic values or clinicopathological features; (3) the expression of CD133 was detected on cancer tissue, rather than in the serum or any other kinds of specimens; (4) the pathological method is the golden standard for diagnosis of lung cancer; (5) articles providing sufficient data to allow the estimation of an odds ration (OR) of OS, DFS and clinicopathological features; (6) articles with no limitations on language or the minimum number of patients of every single study. Reviews, comments, duplicated studies, and irrelevant articles were excluded (Figure 1).

### Data Extraction

The valuable data including author, publication year, country of study, number of patients, detection method, TNM stage, preoperative therapy, clinicopathological features, the number of patients whose cancer tissue was positive for CD133 and the corresponding percentages were extracted from the included papers and illustrated in Table 1. Discrepancies were resolved by discussion among three authors in cases of conflicting evaluations.

**Table 1:** General characteristics of included studies.

Studies	Year	Country	NO. of Patients	Technique	Cutoff values	TNM	Pre-operate therapy	No. of CD133 Positve (%)
Herpel E [26]	2011	Germany	86	IHC	≥20%	I/II	NO	13(15%)
Pirozzi G [27]	2013	Italy	45	FC/IHC/PCR	≥10%	I–III	NO	12(26.7%)
Gottschling S [16]	2013	Germany	55	IHC	≥10%	I/II	NO	10(18%)
Shien K [28]	2012	Japan	50	IHC	>1%	N2–N3	YES	15(30%)
Woo T [5]	2011	Japan	177	IHC	≥17.5%	I	NO	81(45.8%)
Mizugaki.H [1]	2013	Japan	161	IHC	–	I–IV	NO	124(77%)
Alamgeer M [8]	2013	Australia	205	IHC	≥5%	I	NO	104(50.7%)
Xu YH [29]	2010	China	102	IHC	≥10%	I–IV	Not mentioned	51(50%)
Salnikov AV [15]	2009	Germany	81	IHC	≥20%	I–III	Not mentioned	51(63%)
Li F [30]	2011	China	145	IHC	>1%	I	NO	46(31.7%)
Okudela K [2]	2012	Japan	177	IHC	≥17.5%	I	NO	81(45.8%)
Chen JR [25]	2010	China	65	IHC	≥10%	I–III	NO	45(69.2%)
Wei YP [24]	2008	China	77	IHC	≥10%	I–IV	Not mentioned	40(51.9%)
Wu S [31]	2012	China	305	IHC	>1%	II–IV	Not mentioned	149(48.9%)
Cortes-Dericks, L [32]	2012	Switzerland	64	PCR	–	I–III	NO	63(98%)
Bertolini.G [33]	2009	Italy	60	IHC/FC	≥5%	I–IV	–	14(23.8%)
Lin XY [34]	2009	China	54	IHC	>1%	–	–	27(50%)
Zhang HZ [35]	2007	China	77	IHC	≥5%	–	NO	40(51.9%)
Janikova M [9]	2010	Czech	121	TMA/IHC	≥10%	–	–	23(19%)
Sullivan JP [36]	2010	USA	207	TMA/IHC	–	I	–	56(27%)
Li LD [37]	2013	China	50	IHC	–	I–IV	NO	43(85.7%)
Moreira AL [38]	2010	USA	85	IHC	–	I–III	––	18(21.6%)
Tirino V [17]	2009	Italy	89	IHC	–	I–IV	NO	64(72%)

### Statistical Analysis

This study was reported in accordance to the PRISMA-statement (Checklist S1). Statistical analysis was performed by Review Manager 5.0 software. The data was analyzed by means of SPSS version 13.0 (SPSS Inc; Chicago, USA). *P*<0.05 was considered as statistical significance. To minimize heterogeneity, confounding factors, effects of selection, and ascertainment biases across studies, the data extracted from eligible studies were in accordance with the following criteria: (1) the DFS and OS data that were extracted from each eligible study were separately used in the independent analysis; (2) if the study was applied with both univariate analysis and multivariate analysis, the multivariate analysis data were used. Heterogeneity across studies was evaluated by the *Q* test and *P*-values (Cochrans *Q* test, *P*-value>0.05 or *I* ^2^>50% indicated the existence of heterogeneity across studies). Fixed or Random model was used depending on heterogeneity analysis. ORs and RRs were calculated by a fixed-effects model if *P*-value>0.05, otherwise a random effect model was used.

## Results

### Included Studies

Twenty-three studies including 2538 patients met with our criteria in this meta-analysis. The main features of each eligible study were extracted (Table 1). About one-third of the studies dealt with patients with early stage (stage I) of NSCLC, and the rest of the studies was focused on the patients with stage from I to III/IV. Thirteen studies reported that no pre-operative therapy was performed on patients and one study dealing with N2/N3 patients mentioned that pre-operative therapy was applied, while the others had no relevant reports at all. Eleven included studies indicated DFS, 14 studies had OS, and 20 studies reported clinicopathological details. Among the 23 studies, four studies reported neither OS nor DFS, but presented clinicopathological features. In addition, another three articles reported either OS or DFS, but without clinicopathological data.

### Meta Analysis for OS in NSCLC

**Figure 2:**
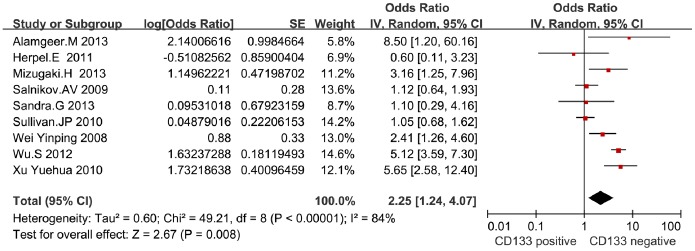
Meta-analysis of overall survival between CD133 positive and CD133 negative in NSCLC patients.

Data of 3-year and 5-year OS extracted from 11 eligible studies were included in the meta-analysis. Since the heterogeneity was significant (*I* ^2^ = 84%, *P* = 0.00001), a random-effect model was used to calculate the OR of OS in NSCLC patients. Meta-analysis found that the patients whose cancer tissue was positive for CD133 expression showed a worse OS than those with negative ones (OR = 2.25, 95% CI: 1.24–4.07, *P* = 0.008, random model), suggesting that CD133 could be an independent prognostic factor in NSCLC patients (Figure 2).

### Meta Analysis on DFS in NSCLC

**Figure 3:**
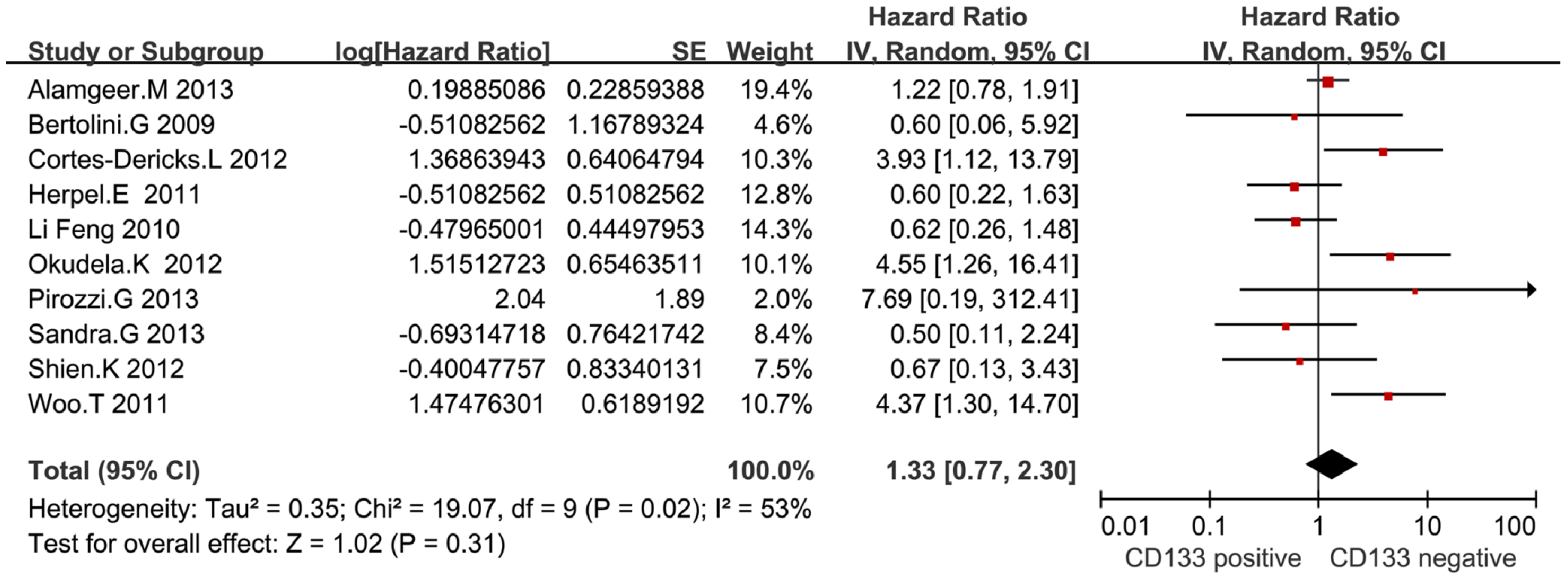
Meta-analysis of disease free survival between CD133 positive and CD133 negative in NSCLC patients.

Data on DFS were also extracted from ten studies. A random-effect model (*I* ^2^ = 53%, *P* = 0.02) was used to calculate the OR of DFS in NSCLC patients. Meta-analysis showed that no difference existed between CD133-positive patients and CD133-negative ones (OR = 1.33, 95% CI = 0.77–2.30, *P* = 0.31, random model). (Figure 3).

### CD133 Expression and Clinicopathological Parameters

Nineteen studies provided the information of various clinicopathological parameters and their correlation with CD133 positive expression was summarized in Table 2. Twelve studies had the statistical data on age of patients. However, none of them reported the association between age and CD133 positive expression. Fifteen studies demonstrated that there was no correlation between CD133 expression and gender. In addition, among nine included studies, only one study showed that smoking was positively correlated with CD133 expression in NSCLC patients.

**Table 2:** Narrative review of the association between clinicopathological parameters and CD133 positive expression with respect to non-small-cell-lung cancer patients.

Items	Significant correlation (*P*<0.05)	Non-significant correlation (*P*≥0.05)
Age	–	[1], [15], [24], [17], [26], [27], [28], [29], [30], [31], [32], [33]
Gender	–	[1], [8], [15], [24], [17], [26], [27], [28], [29], [30], [31], [32], [33], [36], [37]
Smoking	[36]	[1], [8], [24], [30], [29], [28], [32], [33]
Histology	[8], [29], [32], [33], [36], [38]	[1], [15], [24], [17], [25], [26], [27], [28], [30], [31], [35]
Differentiation	[29], [31], [34], [38]	[1], [26], [37], [24], [25], [35]
Lymph node metastasis	[25], [31], [34], [35]	[8], [17], [26], [32], [36]

**Figure 4:**
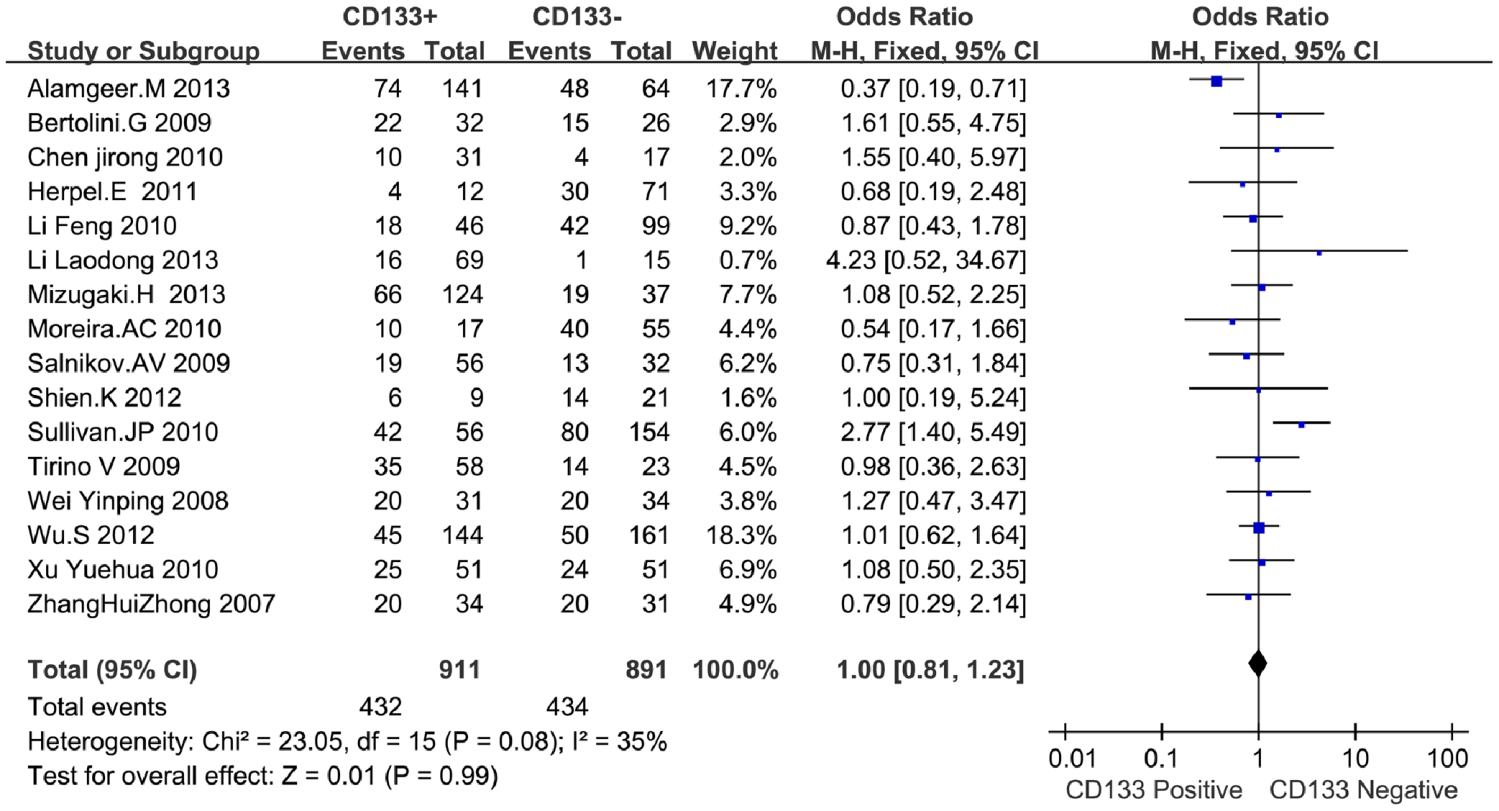
Forrest plot of odds ratios for the association of CD133 expression with adenocarcinoma in NSCLC patients.

**Figure 5:**
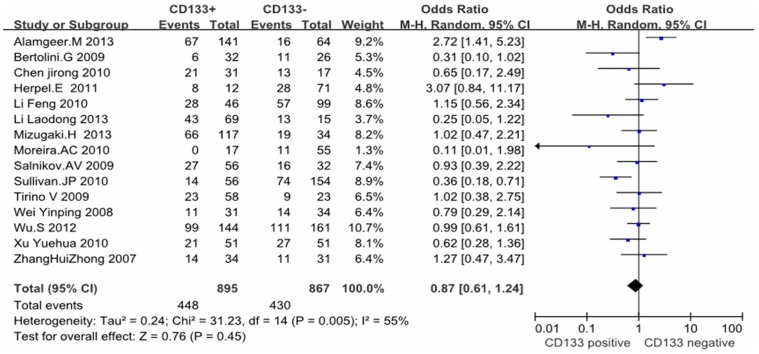
Forrest plot of odds ratios for the association of CD133 expression with squamous carcinoma in NSCLC patients.

**Figure 6:**
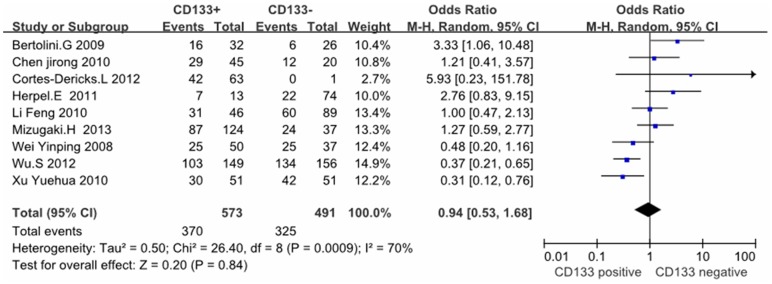
Meta-analysis of correlation between CD133 expression and differentiation in NSCLC patients.

**Figure 7:**
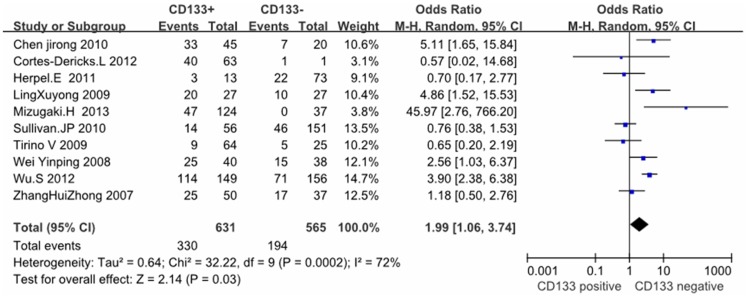
Meta-analysis of correlation between CD133 expression and lymph node metastasis in NSCLC patients.

Except these above-mentioned parameters, controversies also existed on the correlation among histology, differentiation, lymph node metastasis and CD133 expression in these included studies. Seventeen studies evaluated the relationship between CD133 expression and histological types in NSCLC patients. The pooled OR was 1.00, (95% CI = 0.81–1.23, *P* = 0.99, fixed effect) (Figure 4) and 0.87 (95%CI = 0.61–1.24, *P* = 0.45, random-effect) (Figure 5), suggesting that CD133 expression was not associated with the histology of adenocarcinoma or squamous carcinoma. In addition, no correlation was found between the expression of CD133 and the differentiation of NSCLC (OR = 0.94, 95%CI = 0.53–1.68, Z = 0.20, *P* = 0.84, random-effect) (Figure 6). Notably, the meta-analysis suggested that CD133 expression was positively correlated with lymph node metastasis (OR = 1.99, 95%CI = 1.06–3.74, *P* = 0.03, random-effect) (Figure 7).

## Discussion

Up to date, clinically approved biomarkers have been found to guide treatment and predict outcomes in several solid tumors, and several studies have demonstrated that CD133 is a specific cell surface marker of CSCs [1], [2], [18]–[20]. However, the clinical value of CD133 remains controversial in several solid tumors including lung cancer [6]. In this meta-analysis study, we revealed that high expression of CD133 was positively correlated with the poor prognosis of NSCLC patients.

CD133 is a five-transmembrane cell surface glycoprotein, and its gene is specifically located on chromosome 4p15, a region that contains genes related to mature organ homoeostasis, tumorigenesis and cancer progression [19]. Previous studies have determined that CD133-positive cancer cells possess CSCs phenotype, which contributes to the self-renewal and tumorigenic capabilities [21]–[23]. However, controversies remain regarding to whether the correlation of CD133 expression with either poor prognosis or the clinicopathological parameters in NSCLC patients exists [16], [17], [24], [25].

Although our study revealed the positive correlation between CSCs marker CD133 and patients’ survival, CD133 itself as a biomarker has its limitations on predicting prognosis and clinicopathological parameters in patients. Firstly, overall survival (OS) and disease free survival (DFS) were determined from unadjusted RRs in the published papers, and RRs from the survival curves might be less reliable those that from direct analysis of variance. Ideally, measurements should be directly obtained from the statistical data in published papers and then adjusted by using other prognostic factors. Secondly, the condition of patients included in this study was not normalized. Seven included studies were focused on the patients in the early stage, while others dealt with the stage I to IV or the advanced cancer patients. Twelve studies included in this analysis reported that patients did not receive any chemotherapy before surgery and one study targeting at N2–N3 patients reported that neoadjuvant chemotherapy was applied before surgery while others lacked details. Thirdly, the cutoff values of CD133 expression in different papers were variable. Lastly,the OR of each study is generally small and the conclusion might be affected by one or two reports with large ORs. All of these factors might partly influence the significance of CD133 expression in the survival and the clinicopathological analysis.

In summary, the present meta-analysis indicates that CD133 expression is associated with a poor OS and a high rate of lymph node metastasis and no correlation exists between CD133 expression and DFS or other common clinicopathological parameters such as histology and tumor differentiation. CD133 may be a potential prognostic marker and useful therapeutic target in lung cancer, and co-detection of CD133 with other CSC markers including ALDH1A1 and OCT4 may be more valuable and helpful in clinical application in NSCLC patients.

## Supporting Information

Checklist S1 PRISMA checklist.
(DOC)
